# Call up the (cognitive) reserves: how adult socialisation and education influences cognition in the UK Biobank

**DOI:** 10.3389/fpsyg.2025.1542282

**Published:** 2025-06-18

**Authors:** Benjamin Tari, Morgane Künzi, Vanessa Raymont

**Affiliations:** Department of Psychiatry, University of Oxford, Oxford, United Kingdom

**Keywords:** education, leisure activities, built environment, socioeconomic status, UK Biobank, cognition

## Abstract

**Introduction:**

Dementia involves the loss of memory and degradation of cognitive function. Crucially, the onset of dementia may be prevented by identifying and modifying relevant risk factors years before disease onset in midlife. Commonly described modifiable risk factors include social isolation and educational attainment. Here, we aim to understand the relationships between adult activities and their effects on cognition related to mid-life aging in terms of where and how people live.

**Methods:**

We analysed data from the UK Biobank (*N* = 502,165, M_age_ = 56.53, SD_age_ = 8.09, 54.40% female). In particular, our path analysis investigated the associations between years of education in childhood, education later in life, social activities in adulthood, built environment (i.e., coastal distance and percentage of greenspace), socioeconomic status (i.e., Townsend deprivation index), and cognitive functions (i.e., memory, executive function, and abstract reasoning).

**Results:**

Adult education and social activities predict better cognition. Being deprived predicts attendance in adult education classes, but fewer social activities and poorer cognition. Moreover, living in areas with less greenspace and being further away from coastlines predict attendance in adult education classes; however, only greenspace predicts participation in social activities. Finally, less greenspace and further coastal distance support abstract reasoning, whereas further coastal distance predicts poorer executive function.

**Conclusion:**

We demonstrate the potential utility of adult education and social activities which may offset the detrimental effects of deprivation. Accordingly, we argue for improved access to adult social programs in deprived/underserviced areas in the United Kingdom.

## 1 Introduction

Dementia is a syndrome caused by various diseases which damage the brain and subsequently affect an individual’s cognitive function beyond what would be expected during normal aging (Dementia, n.d.). This impairment in cognition is often accompanied by changes to mood, emotional control, motivation, and behaviour. The number of global dementia cases is expected to surpass 150 million by the year 2050 (Nichols et al., 2022). As there is still no cure for dementia, greater emphasis should be placed on means to prevent its onset. Fortunately, a recent Lancet Commission report has described 14 modifiable risk factors which account for ~40% of dementia incidence (Livingston et al., 2024). These include factors such as depression, obesity, smoking and alcohol consumption, social isolation, and education. The latter two account for 11% of the potentially modifiable risk factors and therefore warrant better understanding. For example, the Lancet Commission report describes educational attainment and social isolation as “Early Life” and “Later Life” risk factors, respectively. This is an interesting distinction given the inherent social aspect of formal educational attainment, and the fact that education need not be limited to early age. Literature has previously demonstrated how formal early-life education supports cognition and has a protective effect against the onset of dementia. Indeed, one review found a 7% reduction in dementia risk per additional year of formal education (Xu et al., 2016).

To better understand *how* education influences cognitive performance, our group investigated UK Biobank data via structural equation modelling (SEM) (Tari et al., 2023). Our results predicted associations between formal early life education and indices of deprivation (i.e., the Townsend Deprivation Index), built environment (i.e., coastal distance and percentage of greenspace), and cognition (i.e., pairs-matching, fluid intelligence, the trail-making task B). They demonstrate how higher levels of formal education support multiple domains of cognitive function and that these relationships are directly mediated by deprivation and built environment. These results can be attributed to an accumulation of cognitive reserve which support higher-order functioning in old age (Stern, 2009; Stern et al., 2020, 2023) and provides additional evidence demonstrating the necessity of equitable education. This is particularly relevant given that a review conducted by Lövdén et al. (2020) found that although education was found to be related to cognitive function across the lifespan, the authors suggest that late-life cognition may be primarily influenced by resources gained during *early life*. In contrast, a recent investigation by Takeuchi and Kawashima (2023) demonstrated that participation in formal adult education is also associated with better fluid intelligence performance and a lower risk of dementia onset when compared to those who do not participate in these classes. Taken together, these studies indicate the importance of education in the context of dementia prevention and the preservation of brain health.

As previously alluded to, formal early- and late-life education requires a high degree of social interaction. Recall that the Lancet Commission outlined how education and social isolation account for nearly 11% of the modifiable risk for dementia. Unsurprisingly then, Shen et al. (2022) provided evidence for a negative relationship between social isolation and cognitive performance. This result was further mediated by lower grey matter volumes in isolated individuals. Importantly, social isolation was shown to have a predictive effect on cognitive performance and dementia onset independent of any effects of loneliness. An alternative exploration of *participation* in social activities describes the relationship between social activities and the cognition of Chinese older adults (Fu et al., 2018). This work highlights a unique characteristic of socialisation: a willingness to take part if one is able, and the authors demonstrate how participation in more social activities (i.e., spending time with friends, participating in hobbies, sports, volunteering) relates to various aspects of cognition (Fu et al., 2018). These results can also be attributed to some accumulation and/or maintenance of social reserve important for cognitive reserve.

Stern et al. (2020) define cognitive reserve as an ability to adapt. That is, the ability for one to cope with challenges related to insult or normal day-to-day events. As implied by the term “reserve,” resources which facilitate this adaptability are not required in normal day-to-day activities. Rather, they are accumulated and made available to an individual when necessary. Recently, Stern et al.’ (2023) consensus work has described how cognitive reserve may be influenced by genetic and environmental factors at discrete points, or continuously, across the lifespan. Cognitive reserve proxies include features associated with innate ability and/or accumulated experience, including early age IQ, cognitively stimulating activities throughout life, formal education, occupation, leisure activity, social networks, or other events that might impart cognitive reserve. Accordingly, it is important to tease apart the degree to which specific factors influence cognition. A well-defined aspect of cognition is its natural decline with age (Harada et al., 2013); however, this decline may be mitigated by lifestyle behaviours such as engagement in physical activity, proper sleep habits, and social engagement (Dominguez et al., 2021).

As previously suggested, multiple lifestyle factors may act cumulatively to influence cognitive reserve, and it is the accumulation of these factors which likely impart some protective benefit to cognition. For example, Ihle et al. (2021) demonstrated how a large social network may facilitate the performance of leisure activities which subsequently accumulates cognitive reserve and mitigates cognitive decline. Furthermore, Sauter et al. (2021) analysed self-reported educational levels and the size of individuals’ social network in relation to longitudinal cognitive data in a cohort of older adults. Their results demonstrated that higher levels of education and a larger social network were related to smaller declines in cognitive performance. The above outlines literature which supports the assertion that education and/or adult socialisation are important factors related to the maintenance of cognition. The circumstances which may underpin these relationships are still unknown; however, factors such as socioeconomic status have been implicated (Ihle et al., 2022). Here, we make use of our previously described path analysis which demonstrates relationships between childhood education, indices of deprivation and built environment (RMSEA = 0.06; CFI = 0.94) to investigate how various activities which support cognitive reserve in adulthood influence cognition.

## 2 Materials and methods

### 2.1 Participants

We analysed data from the UK Biobank (Sudlow et al., 2015). In general, the average age of the 502,165 individuals included in our dataset was 56.53 years old and 54.40% were female. As well, the cohort completed on average 18.11 years of early-life structured education (see Table 1 for more detail). The UK Biobank study was approved by the UK Biobank Research Ethics Committee (approval letter dated 17, June 2011: Ref 11/NW/0382) and all data collection procedures were conducted in accordance with the Declaration of Helsinki. All participants gave informed, written consent.

**Table 1:** Participant characteristics, cognitive performance, deprivation, and built environment.

Characteristics	*N*		
Age	502,165	M (SD)	56.53 (8.09)
Sex	502,165	% Female	54.40
Education	495,454	M (SD)	18.11 (2.77)
Adult Education	347,331	% Yes	10.42
Social Score	347,331	M (SD)	1.34 (0.62)
Cognitive function			
Fluid Intelligence	123,539	M (SD)	6.41 (2.06)
Trail-Making Task-B	103,965	M (SD)	66.81 (25.75)
Pairs-Matching Task	118,456	M (SD)	4.20 (3.12)
Deprivation			
Townsend Deprivation Index	501,539	M (SD)	−1.30 (3.10)
Built environment			
Coastal Distance (km)	497,203	M (SD)	41.64(27.71)
Percentage Greenspace	440,549	M (SD)	35.27 (23.22)

Uncorrected group means and standard deviations [M(SD)] for age (in years), years of education, social score, Townsend Deprivation Index score, percentage of greenspace, coastal distance, fluid intelligence score, trail-making task-B duration, and number of incorrect pairs-matching trials. Note that we also present the % of females and the % of individuals who undertook adult education classes in our sample (*N*).

### 2.2 Predictor variables

#### 2.2.1 Built environment, deprivation, social activities, and education

The analyses make use of a model previously described by Tari et al. (2023) and included three main predictor variables: built environment, deprivation and education. Built environment was estimated by participants’ distance to the coastline and the percentage of greenspace around where they lived, whereas the Townsend Deprivation Index (TDI) (i.e., an index of deprivation) reflects one’s “real” living conditions according to geographic constraints (Townsend et al., 2023). To quantify education, the variable “age completed full time education” (variable: 845) was used, and missing data were imputed based on participants’ listed “qualifications” (variable: 6138), which were recoded into their year equivalent. In addition, we sought to include variables describing social behaviours in adulthood, and also included whether participants attended adult education classes. We therefore included the UK Biobank “leisure/social activities” variable (variable: 6160) which asks participants to specify which (if any) of a list of activities (i.e., sports club or gym, pub or social club, religious group, adult classes, and ‘other’ group activity) they attend at least once a week. This was deconstructed into dichotomous variables for each activity and coded as follows: 0 = does not participate in this activity and 1 = participates in this activity. The dichotomous variable “adult education classes” (AdEd) was included in the model, whereas the dichotomous variables of sports club or gym activities, pub or social club activities, religious group activities, and other group activities were summed to create a score reflecting the number of social activities one undertakes in adulthood (Social). We chose to separate adult education and other social activities given our research question which aims to identify the type of adult activities (i.e., leisure/education) which may influence cognitive function in adulthood. As this work looked to build on our previously published model (Tari et al., 2023), only participant age was included in our analysis as a co-variate. These data were collected between 2006 and 2010.

### 2.3 Outcome variables

#### 2.3.1 Cognitive function

As with our previous work, cognitive function was estimated via the incorrect responses to the 6-pair pairs-matching test (PM6; variable: 20132), the time required to complete the trail-making task-B (TMTB) alphanumeric path (variable: 20157), and the score of the fluid intelligence (FI) task (variable: 20191) because they quantify various aspects of cognition including memory, executive function, and abstract reasoning, respectively (see UK Biobank data showcase for more detail; https://biobank.ndph.ox.ac.uk/showcase/) which are vulnerable to cognitive decline (Cornelis et al., 2019). The cognitive variables used here are comprised of data taken between 2014 and 2015.

### 2.4 Statistical analyses

#### 2.4.1 Pre-processing

Data were pre-processed and analysed via Stata SE 17.0 on the Dementias Platform UK (DPUK) Data Portal (Bauermeister et al., 2020). Participants aged 40–73 were included in our analyses to assess the validity of a model which predicts cognitive function across time. As in (Tari et al., 2023), no additional exclusion criteria were applied to our sample. Normality was assessed and variables with skewness values greater than 1.0 were log-transformed to meet normality assumptions prior to subsequent analyses.

#### 2.4.2 Structural equation model

The analysis procedure used here largely corresponds to that which was employed in our previous work, but includes the addition of adult social and education activities which are described above. A more detailed description of data pre-processing and the structural equation model (SEM), as well as the estimation method used (i.e., the Full Information Maximum Likelihood) to handle missing data, can be found in our previous work (Tari et al., 2023). Spearman correlations were computed to explore associations between measures of deprivation, coastal distance, greenspace, the number of incorrect responses on the PM6, the duration of an alphanumeric path in the TMTB, total FI scores, years of education, and age. These results informed the construction of this model which contains the following associations between the variables.

**Figure 1:**
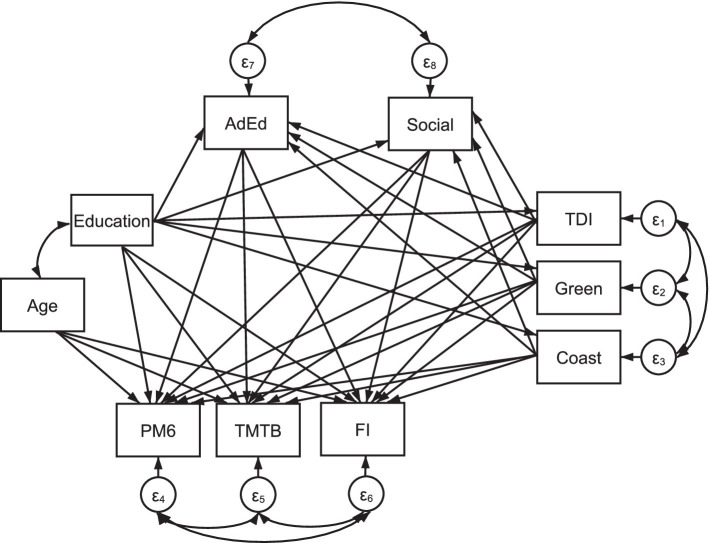
Structural equation model including variables: Education (i.e., imputed years of education), TDI (i.e., Townsend Deprivation Index total score; variable: 22189), Coast (i.e., the distance to the coastline in kilometres; variable: 24508), Green (i.e., percentage of greenspace within a 300 m buffer area; variable: 24503), AdEd (i.e., whether individuals completed adult classes during leisure time; variable: 6160) and Social (a summed score indicating how many of sports club or gym activities, pub or social club activities, religious group activities, and/or other group activities were completed in leisure time; variable: 6160). Age (variable: 21022) was included as a mediator. Three cognitive variables are also included: PM6 (i.e., number of incorrect responses on the pairs-matching task; variable: 20132), TMTB (i.e., the time required to complete the trail-making task-B alphanumeric path; variable: 20157), and FI (i.e., fluid intelligence score; variable: 20191). Covariance links join Age and Education; Social and AdEd; FI, TMTB and PM6; and TDI, Green and Coast. *N* = 502,358.

Education is linked to participation in adult education classes and the number of social activities undertaken, TDI, percentage of greenspace, coastal distance, and cognition (i.e., PM6, TMTB, and FI). TDI, percentage of greenspace, and coastal distance is linked to participation in adult education, the number of social activities undertaken and the three cognitive variables. Finally, participation in adult education classes and the number of social activities undertaken are linked to our chosen variables of cognition. TDI, the percentage of greenspace, and coastal distance; participation in adult education classes and the number of social activities undertaken; and PM6, TMTB, and FI errors terms were allowed to covary, respectively. Figure 1 demonstrates that the variables AdEd and Social are linked as mediators for the relationship between Education, deprivation and built environment with cognition. Due to the large sample size used here, effects were deemed significant when *p* < 0.01 (Lin et al., 2013). Our STATA script is available upon reasonable request for ease of replication.

## 3 Results

### 3.1 Participant characteristics

Participants were 56.53 (SD = 8.09) years of age, comprised of mostly females (54.4%) and had completed a mean of 18.11 (SD = 2.77) years of education (Table 1). As well, 10.42% of participants reported engaging with adult education classes and participants completed an average of 1.34 other social/leisure activities.

### 3.2 Structural equation model

#### 3.2.1 Estimation and fit

The root mean squared error of approximation (RMSEA: differences between predicted and observed outcomes) = 0.06, and the comparative fit index (CFI: metric of the model’s improvement from baseline to proposed iterations) = 0.93. These indices of model fit are considered “good” (Kline, 2016).

**Figure 2:**
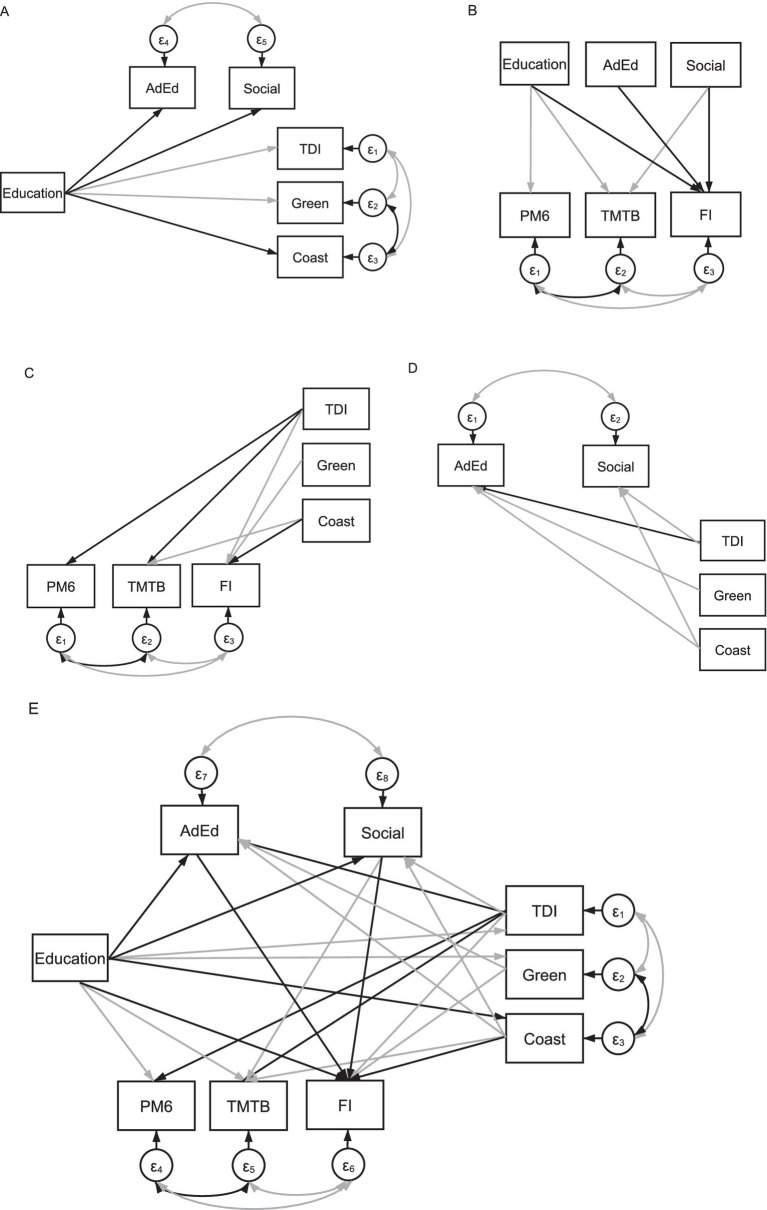
A simplified illustration of our model **(E)** as well as individual panels illustrating direct and indirect links between predictor and outcome variables as well as covariance links. **(A)** Illustrates links between education and social scores (Social), adult education class participation (AdEd), the Townsend Deprivation Index (TDI), greenspace (Green) and coastal distance (Coast). Education, social and adult education scores are linked with the pairs-matching task (PM6), the trail-making task **B** (TMTB) and fluid intelligence (FI) in **(B)**. The Townsend Deprivation Index, greenspace and coastal distance are linked with scores on the pairs-matching task, the trail-making task **B** and fluid intelligence are also included in **(C)**. And in **(D)**, social and adult education scores are linked with the Townsend Deprivation Index, greenspace and coastal distance. Note that only statistically significant positive (black arrows) and negative (grey arrows) interactions are presented.

Figure 2 demonstrates only statistically significant path links within our model and is a direct replication of our previously described work (Tari et al., 2023). That is, lower education was associated with higher deprivation (β = −0.07, *p* < 0.001), living closer to the coast (β = 0.01, *p* < 0.001) and inhabiting an area with more greenspace (β = −0.01, *p* < 0.001); higher deprivation was also associated with living closer to the coast and in greener areas (β > −0.04, *p* < 0.001). Higher education and lower deprivation predicted better cognitive performance [i.e., PM6 (βs = −0.02 and 0.01, *p*s < 0.001), TMTB (βs = −0.17 and 0.08, *p*s < 0.001), FI (β = 0.31 and −0.06, *p*s < 0.001)]. Regarding built environment metrics, living closer to the coastline was associated with poorer cognition [i.e., TMTB (β = −0.01, *p* < 0.001) and FI (β = 0.01, *p* = 0.002)], whereas inhabiting an area with less greenspace was related only to higher FI (β = −0.01, *p* < 0.001). Finally, older age was associated with poorer performance on all cognitive variables (βs > −0.09, *p*s < 0.001).

As described above, our model also incorporates measures of adult education and social activities. Here, our results demonstrate that early-life education is related to the participation in adult education and a higher number of social activities undertaken (βs = 0.10 and 0.06, *p*s < 0.001). In terms of deprivation, being more deprived indicated a higher likelihood that an individual would participate in an adult education class (β = 0.02, *p* < 0.001), but lower deprivation was related to a lower number of social activities undertaken (β = −0.07, *p* < 0.001). Last, living in an area of lower greenspace was predictive of adult education uptake (β = −0.01, *p* < 0.001), but not to the number of social activities undertaken (β = −0.00003, *p* = 0.99) whereas living in areas closer to the coast predicted a higher likelihood of participating in adult education and social activities (βs = −0.01 and −0.01, *p*s < 0.001). As well as early-life and environmental and socioeconomic factors, we examined how adult education and social activity influenced cognitive outcomes in our sample. When describing the effects of adult education and social activities on cognitive performance, our results demonstrate no relationship with PM6 performance (βs = −0.005 and −0.0002, *p*s > 0.13), and that adult education was not related to TMTB completion time (β = 0.003, *p* = 0.40). In contrast, fewer social activities predicted slower TMTB completion time (β = −0.01, *p* = 0.002). Finally, better FI was related to undertaking adult education and participating in more social activities (βs = 0.01 and 0.01, *p*s < 0.001). Our full SEM output is presented in Table 2.

**Table 2:** Structural equation model output.

	Predictor	β	SE	z	*p*	95% CI	
TDI							
	Education	−0.07	0.001	−51.32	<0.001*	−0.08	−0.07
Coast							
	Education	0.01	0.001	9.16	<0.001*	0.01	0.02
Green							
	Education	−0.01	0.002	−5.20	<0.001*	−0.01	−0.005
AdEd							
	TDI	0.02	0.002	8.54	<0.001*	0.01	0.02
	Coast	−0.01	0.002	−7.05	<0.001*	−0.02	−0.01
	Green	−0.01	0.002	−3.76	<0.001*	−0.01	−0.003
	Education	0.10	0.002	61.42	<0.001*	0.10	0.11
Social							
	TDI	−0.07	0.002	−36.51	<0.001*	−0.07	−0.06
	Coast	−0.01	0.002	−3.71	<0.001*	−0.01	−0.003
	Green	−0.00003	0.002	−0.02	0.99	−0.004	0.004
	Education	0.06	0.002	35.00	<0.001*	0.06	0.06
PM6							
	TDI	0.01	0.003	3.66	<0.001*	0.01	0.02
	AdEd	−0.005	0.003	−1.50	0.13	−0.01	0.001
	Social	−0.0002	0.003	−0.05	0.96	−0.01	0.01
	Coast	0.01	0.003	1.97	0.05	0.00002	0.01
	Green	−0.002	0.003	−0.52	0.60	−0.01	0.004
	Age	0.13	0.003	43.01	<0.001*	0.12	0.14
	Education	−0.02	0.003	−5.13	<0.001*	−0.02	−0.01
TMTB							
	TDI	0.08	0.003	24.72	<0.001*	0.07	0.08
	AdEd	0.003	0.003	0.85	0.40	−0.003	0.01
	Social	−0.01	0.003	−3.09	0.002*	−0.02	−0.004
	Coast	−0.01	0.003	−5.27	<0.001*	−0.02	−0.01
	Green	0.002	0.003	0.73	0.47	−0.004	0.01
	Age	0.38	0.003	146.91	<0.001*	0.38	0.39
	Education	−0.17	0.003	−57.92	<0.001*	−0.18	−0.17
FI							
	TDI	−0.06	0.003	−21.09	<0.001*	−0.07	−0.06
	AdEd	0.01	0.003	4.66	<0.001*	0.01	0.02
	Social	0.01	0.003	4.87	<0.001*	0.01	0.02
	Coast	0.01	0.003	3.04	0.002*	0.003	0.01
	Green	−0.01	0.003	−4.24	<0.001*	−0.02	−0.01
	Age	−0.09	0.003	−30.94	<0.001*	−0.09	−0.08
	Education	0.31	0.003	115.17	<0.001*	0.31	0.32

The left most column indicates the variables of interest and include: TDI (i.e., Townsend Deprivation Index), Coast (i.e., the distance to the coastline in kilometres), Green (i.e., percentage of greenspace within a 300 m buffer area), AdEd (i.e., participation in an adult education class), Social (i.e., sum of participation in sport, pub, religious and other group activities), PM6 (i.e., number of incorrect responses on the 6-pair pairs-matching task), TMTB (i.e., the time required to complete the trail-making task-B alphanumeric path) and FI (i.e., fluid intelligence score). Predictor variables indicate those which have direct paths to the variable of interest. The output provides standardised beta (β) values, standard error (SE), z scores, p values, and 95% confidence intervals (95% CI). For ease of interpretation, *p* values marked with * indicate a statistically reliable association: *p* < 0.01. *N* = 502,165.

## 4 Discussion

Our work aimed to explore how adult education and social activities influence cognition in a middle-aged cohort: UK Biobank. Our results indicate that age, early life education, deprivation and built environment influence cognition in a manner similar to that which has been previously defined by our group (Tari et al., 2023). These associations are described elsewhere and will therefore not be re-addressed here. Rather, the below will outline how early life education, deprivation, and built environment impact adult education and social activities which in turn influence cognitive function.

### 4.1 Early-life education, deprivation, and built environment influence adult education and social activity uptake

Our model indicates that individuals who complete more education early in life are more likely to participate in social activities and undertake adult education classes. Results also demonstrate how individuals who are more deprived are more likely to participate in adult education but not in other social activities. Last, living in an area of lower greenspace is related to a higher likelihood of participating in adult education, but not social activities, whereas living nearer a coast predicted participation in both.

Early-life education naturally involves a set curriculum which prepares students to progress in education and workplace scenarios (Christison, 2013). As well, a formal education setting provides individuals with an environment to practice socialisation behaviours (Phillips and Lowenstein, 2011). This combination allows for individuals to advance in terms of socioeconomic status and social capital (Menardo et al., 2022). For example, higher education is associated with better income and a higher likelihood of achieving a better socioeconomic status (Aikens and Barbarin, 2008). A longer period of time spent in educational settings also affords individuals with the opportunity to practice social behaviours which persist into adulthood; such as the ability to follow instructions, set goals, and solve problems. Similarly, they benefit in terms of character, skill and social development (Christison, 2013). Those exposed to an education system with access to a high number of socialisation opportunities are also more likely to contribute positively to their communities (Barber et al., 2013). The cumulative experiences gained through these social interactions are likely to increase one’s motivation to commit to social activities in the future. If we consider the links between deprivation and our chosen adult education and social activities, we see that higher education is likely to predict lower deprivation, and that lower deprivation subsequently predicts more social activities (i.e., sports club or gym, pub or social club, religious group, ‘other’ group activity) and less adult education participation. An individual in this deprived situation may have only a finite amount of free time with which to engage in additional activities. It is conceivable that when presented with this choice, highly deprived individuals may seek opportunities to increase their social capital and improve their status (Im and Shane, 2022). Adult education has been shown to have a wide range of benefits, including employability for less advantaged individuals (Winterton and Winterton, 2003). Unfortunately, adult education in England has traditionally been more easily accessible for affluent individuals (McGivney, 2001). Moreover, a report by the British Social Mobility Commission reveals the striking disparity in social participation depending on individuals’ socioeconomic background. The report focussed primarily on young people; however, as youth socioeconomic and deprivation levels are related to those in adulthood (Duncan and Magnuson, 2012), these results can likely be applied across ages. Results demonstrated that those individuals belonging to wealthier families were much more likely to participate in a wide range of extracurricular activities (e.g., sport, clubs, volunteer work) which facilitate crucial social and cognitive skills. These behaviours are likely to continue into adulthood and account for the likelihood with which individuals participate in adult education and social activities (Christison, 2013). Finally, one’s immediate environment also plays a crucial role in the ability/willingness to undertake adult activities. Social expenditure in in the United Kingdom is often lower rural and coastal areas than it is in wealthier regions (Asthana and Gibson, 2022). One would conclude then, that those living in these regions would be less likely to partake in any adult activities. On one hand, our results do support this assertion as those with higher levels of deprivation are more likely to live in areas with less greenspace. When coastal distance is taken into account, however, we find that being closer to the coast facilitates participation in both types of activities. In terms of adult education this is unsurprising as the above outlines how those with lower socioeconomic status and those with less early life education may strive to improve their qualifications via adult education classes. It may be that this engagement in adult education stimulates the need for additional social participation for these individuals. Regardless, this is a result which warrants future investigation.

### 4.2 Adult education and social participation differentially influence cognitive function

Our model investigated how proxies of cognitive reserve accumulated in adulthood may support mid-life cognitive performance. We found that, in contrast to early-life education, adult education and social activities differentially support cognitive function: participation in adult education was only related to improved fluid intelligence; more social activities were related to both TMT and fluid intelligence performance. In contrast, performance on the PM6 was unaffected by either adult education or social activities. To address this, we first outline the individual cognitive domains assessed by our chosen cognitive tests.

#### 4.2.1 Memory performance is not related to adult education or socialisation behaviours

The PM6 task is an assessment of memory and tasks such as those used here have been previously shown to be unrelated to adult education uptake (Takeuchi and Kawashima, 2023). The authors proposed that this null effect might be related to the curriculum of adult education classes in question, which were not controlled for here, and which may not emphasise rote memorisation, but the application of taught information. This further emphasises the cognitive-domain-specific effects of adult education. In contrast, that adult social behaviours do not affect memory performance is somewhat surprising given previous work has demonstrated that participation in social activities is related to better episodic memory performance (Hu et al., 2012). These results may be due to the different categorisations of social activities described by Hu and colleagues. However, although the authors categorise activities differently to UK Biobank, their model also considers the sum of social activity scores and thus makes this explanation unlikely. A more parsimonious explanation for this discrepancy may be the cultural differences between our cohorts (i.e., UK Biobank vs. China Health and Retirement Longitudinal Study). For example, the authors highlight how in Asian populations, older adults individuals are more likely to reside with family compared to those living in western societies. Thus, social activities for individuals in China may serve different purposes to those living in the United Kingdom. In any case, future investigation of this discrepancy with a particular focus of cultural differences is warranted.

#### 4.2.2 Executive function and fluid intelligence are moderated by social behaviours and education uptake

When considering effects of adult education and social activities on executive function and fluid intelligence performance, we see further evidence for domain- as well as activity-specificity. In terms of social activities, our results are in keeping with similar work by Ihle and colleagues (Ihle et al., 2019). The authors performed cross-lagged analyses on the relationship between leisure activity and cognitive performance (i.e., TMT). Results indicated that more leisure activities predicted better TMT performance even at a follow-up period conducted 6 years after baseline. In terms of fluid intelligence, Takeuchi and Kawashima (2023) provide evidence that adult education classes are associated with retention of fluid intelligence 5 years after baseline. Moreover, social engagement defined as participation in social outings, exercise, religious activities, as well as intellectual engagement are positively related to better fluid intelligence in a similar British cohort (i.e., Cambridge Centre for Aging and Neuroscience) (Borgeest et al., 2020). Our results and those of the existing literature can be explained by the cognitive reserve hypothesis (Stern et al., 2020, 2023). Cognitive reserve is influenced by one’s environment, and is accumulated via education and participation in leisure-time activities. Life course trajectories of normal cognitive aging are likely to be influenced by how much reserve an individual has amassed in their lifetime [for review see (Cullati et al., 2018)]. Similarly, the absence of adequate cognitive reserves (i.e., vulnerability) may expose individuals to a higher risk of cognitive decline. The inability to cope with, and/or recover from, stressors, and individuals characterised as vulnerable makes them more likely to suffer adverse health effects in later life (Cullati et al., 2018).

### 4.3 Implications

As demonstrated above, early life education supports cognitive function; adult education and socialisation activities are predicted by one’s sociodemographic status and environment; and the type of activity one undertakes in adulthood differentially supports specific cognitive domains. Our results reinforce the need to provide equitable access to education in early life and increase investment in deprived areas. A more detailed case for this can be found in our previous work (Tari et al., 2023). The above focuses mainly on the benefits of adult education and socialisation behaviours and emphasises the ability for these activities to offset the negative effects of higher levels of deprivation. Accordingly, the importance of social programs in these regions is vital. They may have noticeable effects on individual cognitive health and/or have a positive feedforward effect for the community in general (Christison, 2013). Alternatively, due to the strong relationship between early life education and indices of deprivation, cognition, and social behaviours, it may be that investment in early-life education may be a more prudent means to best support the community at large. Of course, more research is necessary before changes to related policy can be suggested.

### 4.4 Limitations and future directions

We recognise that our work is limited in several methodological aspects. First, our model is cross-sectional and therefore cannot provide information regarding the causality (although the predictors were measured before the outcomes), as well as the potential long-term effects of adult education and social activities, nor the long-term effect on dementia incidence. Moreover, the magnitude of these effects cannot be directly compared to early-life education. Previous work has investigated the long-term effects of adult education; however, similar work should be done regarding social behaviours. Second, our model has no indices of childhood socialisation activities and we therefore cannot comment on the similarities or differences between adult and childhood behaviours. Moreover, the indices of social activity and adult education which we do present are binary variables which were summed to create a composite score. Therefore, we are unable to distinguish which activity best explains our results. Third, our study makes use of data in the UK Biobank; a cohort of over 500,000 individuals. Despite being one of the more detailed datasets in the world, the sample is inherently skewed. This cohort is comprised of a single, highly educated, ethnically homogenous British group which will likely not be generalizable to other global cohorts. Accordingly, future studies should be conducted in other, potentially more diverse datasets. Finally, an interesting avenue which warrants further investigation is the apparent distinction between the utility of the “type” of cognitive reserve activity present in our results. It may be that one type of activity (e.g., social) provides more cognitive reserve-related benefits than any other, and understanding this would be useful when steering discussions on policy change.

### 4.5 Conclusion

Our work demonstrates how adulthood education and social participation may directly affects specific cognitive domains, as well as how these behaviours are influenced by individual-specific indices of early-life education, deprivation and built environment. Accordingly, we provide evidence for the need to promote and improve access to adult social programs in deprived/underserviced areas in the United Kingdom.

## Data Availability

Publicly available datasets were analyzed in this study. This data can be found at: https://portal.dementiasplatform.uk/.
